# The Application of Computer Musculoskeletal Modeling and Simulation to Investigate Compressive Tibiofemoral Force and Muscle Functions in Obese Children

**DOI:** 10.1155/2013/305434

**Published:** 2013-10-31

**Authors:** Liang Huang, Jie Zhuang, Yanxin Zhang

**Affiliations:** ^1^Department of Sport and Exercise Science, The University of Auckland, Auckland 1072, New Zealand; ^2^School of Kinesiology, Shanghai University of Sport, Shanghai, China

## Abstract

This study aimed to utilize musculoskeletal modelling and simulation to investigate the compressive tibiofemoral force and individual muscle function in obese children. We generated a 3D muscle-driven simulation of eight obese and eight normal-weight boys walking at their self-selected speed. The compressive tibiofemoral force and individual muscle contribution to the support and progression accelerations of center of mass (COM) were computed for each participant based on the subject-specific model. The simulated results were verified by comparing them to the experimental kinematics and EMG data. We found a linear relationship between the average self-selected speed and the normalized peak compressive tibiofemoral force (*R* ^2^ = 0.611). The activity of the quadriceps contributed the most to the peak compressive tibiofemoral force during the stance phase. Obese children and nonobese children use similar muscles to support and accelerate the body COM, but nonobese children had significantly greater contributions of individual muscles. The obese children may therefore adopt a compensation strategy to avoid increasing joint loads and muscle requirements during walking. The absolute compressive tibiofemoral force and muscle forces were still greater in obese children. The long-term biomechanical adaptations of the musculoskeletal system to accommodate the excess body weight during walking are a concern.

## 1. Introduction

The prevalence of obesity among children has increased dramatically in the past few decades, and excess body weight during childhood was found to be indicative of skeletal problems in later life [1–4]. Activities of daily living such as walking and stair climbing impose relatively large loads and movements on weight-bearing joints in obese children [5, 6]. Abnormal loading can have adverse effects on joint health, resulting in more discomfort or pain of the musculoskeletal system [7, 8]. Previous *in vivo* studies have also shown that excessive compressive forces may damage articular cartilage and lead to joint osteoarthritic changes [9, 10]. Since obesity is a known risk factor for musculoskeletal pain and disorders [11], determining the differences in knee joint loads between obese and nonobese children may contribute to clarifying the pathophysiologic role of obesity in the development and progression of knee problems (e.g., knee osteoarthritis). In addition, knowledge of individual muscle activity during movement could improve the diagnosis of the  obese individual with potential gait abnormalities in terms of joint loading and muscle function.

Traditional gait analysis using inverse dynamics is limited by its ability to create an integrated understanding of muscle activities and joint movements [12]. The joint kinetics computed from the equations of motion are the net value. The results represent the force of all muscles crossing a joint, but the musculoskeletal system is mechanically redundant. The information about the cocontraction of muscles and the biauricular muscle activities is not available. Electromyography (EMG) provides important data to support the inverse dynamic analysis for the estimation of joint moments. However, an EMG signal just represents the summed effects of the activity of a group of muscles; there are still no estimates of individual muscle forces [13]. Since it is far more difficult to invasively obtain tissue stresses and muscle forces *in vivo*, computational modeling and simulation are recognized as a vital complementary tool to estimate multiple variables of interest under dynamic conditions [12]. In recent decades, a large number of simulation studies have been developed to investigate the causal relationship between muscle force and joint movement during walking [14]. The information was integrated with joint kinematics to determine the corresponding forces and stresses acting on the bones. Liu et al. [15] reported muscle contributions to support and accelerate body COM over different walking speeds in eight children. Steele et al. [16] examined how muscle forces and compressive tibiofemoral force change with the increasing knee flexion associated with crouch gait in cerebral palsy children. Unfortunately, no study has investigated the effects of obesity on joint reaction force and individual muscle activity using musculoskeletal simulations.

Therefore, the purpose of this study was to utilize musculoskeletal modeling and forward simulation to investigate the gait strategy of obese children at the musculoskeletal level. We simulated the tibiofemoral force to investigate the relationship between obesity and knee joint loading. By analyzing individual muscle function, we compared the mechanisms of how individual muscles contribute to the support and progression accelerations of COM and joint kinematics between obese and nonobese children during normal walking.

## 2. Materials and Methods

### 2.1. Experimental Data

The three-dimensional kinematic and kinetic data were collected from eight obese (the OB group) and eight normal-weight (the NW group) male children aged 8–12, walking at their self-selected speed over the ground. All participants were recruited by advertisements placed in the local communities. Body mass index (BMI) was used to classify all participants according to the  age- and gender-specific cut-off points for obesity and normal as defined by Cole et al. [17]. The demographics of all participants are represented in Table 1. A 16-camera Vicon motion analysis system (Oxford Metrics, Oxford, UK) was used to collect three-dimensional positions of 27 markers in the static trial and 23 markers in the walking trial at 100 Hz based on the Cleveland Clinic marker set. Ground reaction forces were recorded from two force plates at 1000 Hz. Experimental data were preprocessed in Vicon Nexus (Oxford Metrics, Oxford, UK). All children and their guardians read and signed an informed consent form approved by the local Human Research Ethics Committee.

### 2.2. Musculoskeletal  Model

This study used a 3D  generic  musculoskeletal  model built  in OpenSim (https://simtk.org/) v3.0 software [18], with 23 degrees of freedom (DOF) and 92 Hill-type muscle-tendon actuators [19]. A ball-and-socket joint was used to represent the hip and pelvis to trunk joints (3 DOF). The knee joint (1 DOF) was modeled as a planar joint in the flexion/extension axis [20]. Each ankle was modeled as a revolute joint (1 DOF). This musculoskeletal model has been previously used for studies involving healthy children and children with cerebral palsy [15, 16]. 

### 2.3. Simulation

The generic model was scaled to each subject according to the position of anatomical reference points. Inverse kinematics were applied to calculate the joint kinematics (joint angles and translations) over a gait cycle. According to the kinematics and measured ground reaction force, the equation of motion in this dynamic system was applied to calculate the forces and moments at each joint. A static optimization algorithm decomposed the net joint moments into individual muscle forces by solving an optimization problem that minimized the sum of the squares of the muscle activations. Then, the residual reduction algorithm was used to make the data of the joint kinematics more dynamically consistent with the experimental ground reaction force data. The next step involved using Computed Muscle Control (CMC), which found the muscle excitations that drove the models to track the desired kinematics [18]. Simulated joint kinematics were compared to the measured kinematics data to make sure that the simulations were able to track the experimental data. In order to evaluate the accuracy and validity of the musculoskeletal simulations,   the  total  simulated COM accelerations due to each force were compared to the measured COM acceleration. The simulated muscle activations were also compared to the  EMG data from 85 normal children aged 10.5 ± 3.5 as reported by Schwartz et al. [21].

### 2.4. Compressive Tibiofemoral Force

The compressive tibiofemoral force represented the sum of contact forces between the tibial and femoral cartilage and all ligament forces crossing the tibiafemoral joint. It was calculated by the joint reaction analysis algorithm in OpenSim software, which incorporates a postprocessing procedure that uses the muscle forces and joint kinematics to calculate the resultant joint loads. The Newton Euler equation is

(1) $$\begin{array}{c} {\underline{R}}_{\text{knee}}=M_{T}\left(\underline{q}\right)\ddot{\underline{q}}-\left[{\underline{F}}_{M}+G_{T}\left(\underline{q}\right)+{\underline{R}}_{\text{ankle}}\right], \end{array}$$

where $M_{T}(\underline{q})\;$is the mass matrix of a tibia (6 × 6).$\;\underline{q}$ represents the vector of angular and linear displacement and $\underline{\ddot{q}}$ represents the vector of angular and linear acceleration of the tibia. ${\underline{F}}_{M}$ are the muscle forces and moments required to reproduce the knee joint movements of each subject throughout the gait cycle, which were obtained from static optimization results. $G_{T}(\underline{q})$ represents the gravitational loading. ${\underline{R}}_{\text{ankle}}\;$represents the ankle reaction force and moment. The calculation details have been previously described by Steele et al. [16]. The muscle forces were obtained from static optimization algorithm in OpenSim. Three major muscle groups across the knee (quadriceps, hamstrings, and gastrocnemius) were analyzed to investigate the relationship between the muscle forces and the compressive tibiofemoral force.

### 2.5. Muscle Function Analysis

Static optimization is computationally efficient and has been widely used in the estimation of muscle forces and joint reaction forces [14]. However, it cannot provide data of individual muscle contributions to the acceleration of COM [13]. It is necessary to use forward dynamics to extend the modeling analysis for predicting gait adaptation. Thus, an induced acceleration analysis in OpenSim was used to compute the contributions of individual muscles to vertical and fore-aft COM accelerations over a complete gait cycle. Based on the muscle excitation level from CMC results, the motion was simulated forward over a short-time interval (0.01 s) to calculate the resulting change in the model's COM [22]. For each muscle, its induced acceleration was integrated over the gait cycle and defined by its contribution to COM [22]. The individual muscle contribution ${\ddot{\underline{q}}}_{m}$ was formulated as follows:

(2) $$\begin{array}{c} {\ddot{\underline{q}}}_{m}={\left[M\left(\underline{q}\right)\right]}^{-1}R\left(\underline{q}\right){\underline{F}}_{m}, \end{array}$$

where$\;\;M(\underline{q})\;$is the mass matrix of COM (6 × 6). $R(\underline{q})$ is a diagonal matrix of muscle moment arms (6 × 6). ${\underline{F}}_{m}$ is a vector of muscle force. The muscle forces obtained from CMC were used in this equation to estimate the individual muscle contribution to COM, while the muscle forces obtained from static optimization were used to calculate joint loads. The results of static optimization and CMC were similar in their determination of muscle forces in normal walking [12].

To simplify data analysis, the forces and contributions of the actuators performing similar functions were summed. The quadriceps forces and contributions were the sum of the rectus femoris, the vastus medialis, the vastus intermedius, and the vastus lateralis. The hamstring muscle group included the semimembranosus, semitendinosus, biceps femoris long head, and biceps femoris short head. The gastrocnemius was the sum of medial and lateral gastrocnemius forces. Contributions from the gluteus maximus superior middle and inferior muscles and gluteus medius anterior, middle, and posterior muscles were summed into one gluteus maximus and one gluteus medius contribution, respectively. Vasti contribution was computed by the sum of the vastus medialis,   vastus intermedius, and vastus lateralis. A dorsiflexor contribution is composed of contributions from tibialis anterior, extensor halluces longus, extensor digitorum longus and peroneus tertius.

### 2.6. Data Statistics

Means and standard deviations of temporal-spatial gait parameters, peak compressive tibiofemoral force, and muscle forces were calculated for each group. An independent *t*-test was performed to compare the  differences between the OB group and the NW group. A linear regression analysis was performed to identify the relationship between walking speed and peak compressive tibiofemoral force for each subject, and the correlation coefficient value (*R* ^2^) was calculated. *P* < 0.05 indicated statistical significance.

## 3. Results and Discussion

### 3.1. Validity of the Simulated Results

**Figure 1:**
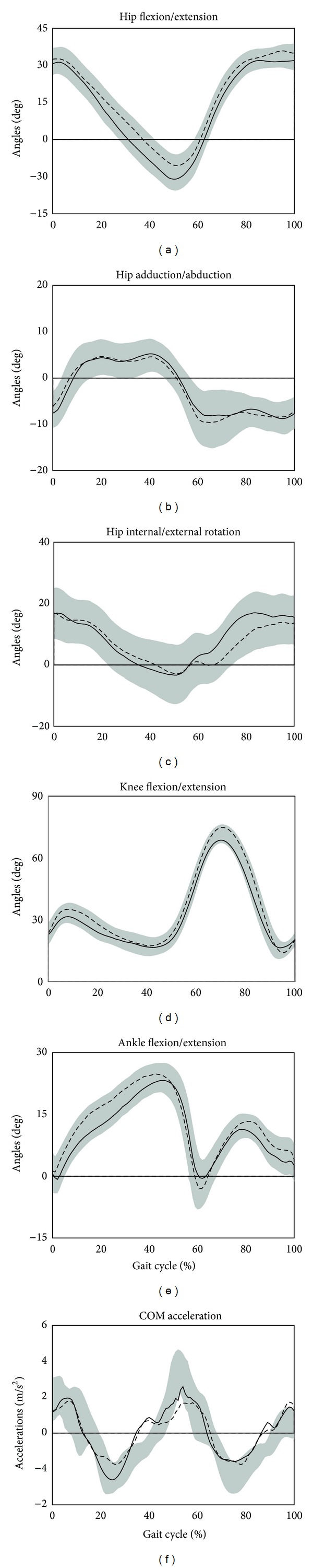
Kinematics tracking results of the comparison between simulated and measured results. The shaded areas represent the mean ± one standard deviation of measured kinematics data. The solid lines represent the mean simulated kinematics for the OB group, while the dotted lines represent the mean simulated kinematics for the NW group.

**Figure 2:**
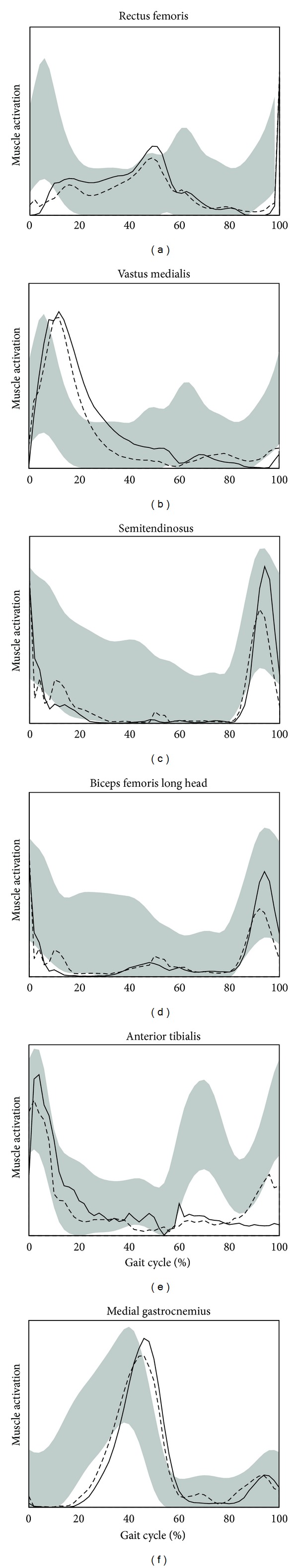
Qualitative comparisons of simulated muscle activity and experimentally measured EMG. The shaded areas represent the mean ± one standard deviation of EMG data reported by Schwartz et al. [21]. The solid lines represent the mean simulated muscle activations for the OB group, while the dotted lines represent the mean simulated muscle activations for the NW group.

The  joint angles were averaged from the simulation over eight participants with three walking trials each for the OB and NW group, respectively. Simulated joint kinematics were able to track the measured kinematics data, and the vertical COM acceleration also matched well with experimental data, as shown in Figure 1. In addition, the joint angles were consistent with the previous experimental-based studies [23, 24]. The simulated muscle activation data were qualitatively compared to the EMG data reported by Schwartz et al. [21]. Simulated rectus femoris, vastus medialis, semitendinosus, biceps femoris long head, and medial gastrocnemius were highly consistent with the experimentally measured EMG data (Figure 2). Unlike what Schwartz et al. [21] reported,   the anterior tibialis in our simulation did not exhibit a burst of activation at push-off and the end of swing phase. However, there was still a slight activation during this time. Therefore, the use of subject-specific modeling allowed to us obtain the agreement between the experimental and simulated data.

### 3.2. Compressive Tibiofemoral Force

**Figure 3:**
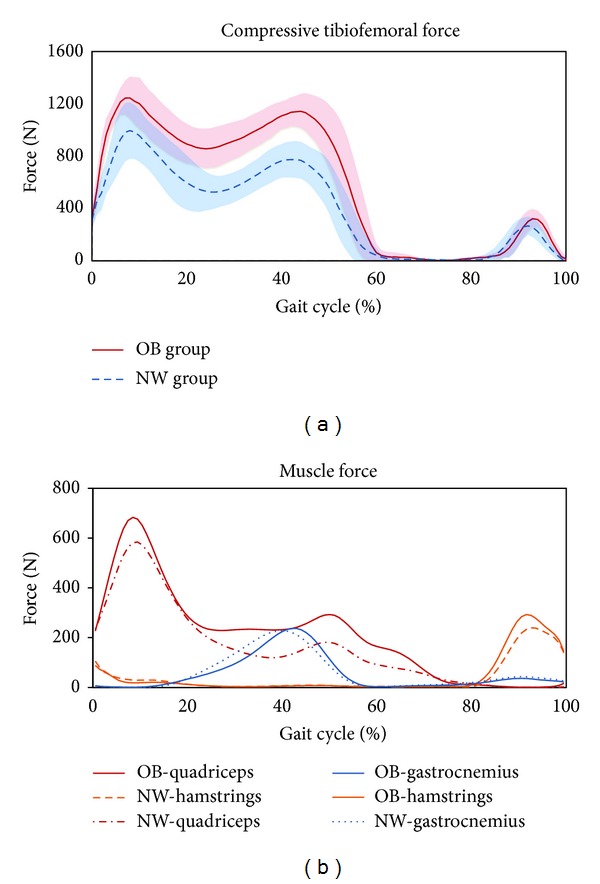
(a) Represent the compressive tibiofemoral force over a gait cycle during normal walking. The solid lines represent the mean compressive tibiofemoral force for the OB group, while the dotted lines represent the mean compressive tibiofemoral force for the NW group. (b) Represented the forces of three major muscle groups acting at the knee.

**Figure 4:**
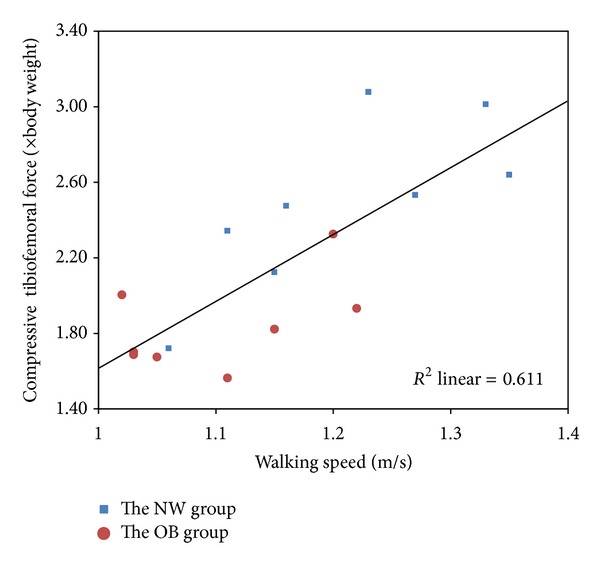
Correlation of self-selected walking speed with peak compressive tibiofemoral force.

The results showed that the absolute compressive tibiofemoral force was significantly higher in obese children over the gait cycle due to the excess body mass (Figure 3). The association between obesity and joint load during walking is intuitive. Even though the obese children walked at a significantly slower speed, the absolute compressive tibiofemoral force was still 25% higher than that in normal-weight children. After body weight normalization, the peak value of compressive tibiofemoral force was significantly higher in the NW group than that in the OB group (Table 2). There was a linear relationship between the average self-selected speed and the normalized peak compressive tibiofemoral force (*R* ^2^ = 0.611). The relationship is described by

(3) $$\begin{array}{c} F_{\text{knee}}=3.585\times V_{\text{walking}}-1.975, \end{array}$$

where *F*_knee_ is the peak compressive tibiofemoral force normalized by body weight and *V*_walking_ is the most comfortable walking speed selected by subject (Figure 4). 

The peak value of compressive tibiofemoral force appeared at the contralateral toe-off following the first heel strike with a magnitude of approximately 2.5 times the body weight (BW) in normal children and 1.8 times the BW in obese children (Table 2). These findings are within the range of previously reported *in vivo* knee contact force measurements made by instrumented implants during overground walking, showing that the peak value of compressive tibiofemoral force ranged from 2.1 to 3.0 BW [25]. Within the limited studies in the obese population, Messier et al. [26] found 3.1 times BW of compressive tibiofemoral force in 142 obese elderly persons with knee osteoarthritis. Aaboe et al. [27] reported 2.7 times BW of peak knee loadings during walking in a similar population. These results were considerably higher than ours. However, both of the studies focused on the obese adults with knee disorders. No *in vivo* experimental data or computational data were available for obese otherwise healthy children.

According to the muscle force results (Figure 3), we were able to analyze how muscle forces act on the joints over a gait cycle. In agreement with previous the studies [16, 28, 29], our results showed that the total joint compressive force had two peaks. The first peak appeared at the contralateral toe-off following the initial heel strike, which was mainly caused by the activity of the quadriceps. The second peak was slightly lower than the first peak, presenting at contralateral heelstrike before toe-off. It resulted from the force developed by both the gastrocnemius and quadriceps. There was no significant difference in the magnitude of the gastrocnemius force between obese and nonobese children at the stance phase. In the OB group, quadriceps contributed the most to joint loading throughout the stance phase, and the contribution of the quadriceps and gastrocnemius was almost the same at the second peak. In contrast, nonobese children used more gastrocnemius force than quadriceps force at the late stance phase. The difference between quadriceps forces at this period partly explained a higher second peak in the OB group. The action of hamstrings also contributed to the knee joint reaction force but only appeared in the early stance before contralateral toe-off and the end of swing phase. Therefore, while more quadriceps forces were needed by the obese children to support the body weight, more compressive tibiofemoral force would be acting on the knees.

### 3.3. Muscle Function

Induced acceleration analysis was used to determine the relationship between an isolated change in a muscle force and the corresponding changes in the movement [14]. Based on forward dynamics solutions, we calculated the contribution of individual muscles to the vertical (support the body) and forward (progression) COM acceleration. 

**Figure 5:**
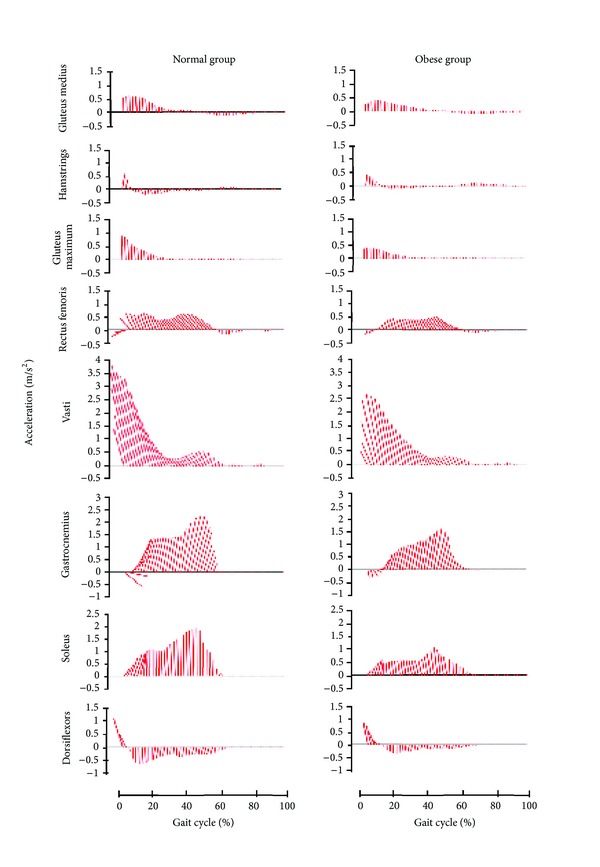
Muscle contributions to the acceleration of COM between obese and normal-weight children during self-selected speed walking. Each ray is the resultant vector of the vertical and fore-aft accelerations, averaged across eight subjects.

The results showed that muscle coordination appeared to be invariant to the differences in body mass between groups. Specifically, hip extensors (gluteus maximus and hamstrings), gluteus medius, knee extensors (rectus femoris and vasti), and ankle dorsiflexors were active in early stance to serve the function of providing support in both groups. This activity explained the appearance of the first peak in the vertical COM acceleration in early stance. The results also confirmed that quadriceps, especially vasti, generated the majority of support and decelerations in the first half of stance, which caused the first peak of compressive tibiofemoral force. In the late stance phase, gastrocnemius and soleus, which are the primary muscles for plantar flexion, contributed most to the vertical and forward COM acceleration. The gluteus maximus and gluteus medius play only a minor role in generating forward acceleration during walking. Clearly, the ankle plantarflexors provided most of the fore-aft force for continued progression for both obese and nonobese children. The hamstrings also contributed to the acceleration of the COM throughout the stance phase, but the magnitude was relatively small (Figure 5). It is not surprising that obese children use similar muscles to support and accelerate body COM, because there were no neuromuscular impairments or diagnosed misalignment syndrome in these obese children. These results were in broad agreement with similar studies for walking at the self-selected (free) speed [15, 30].

**Figure 6:**
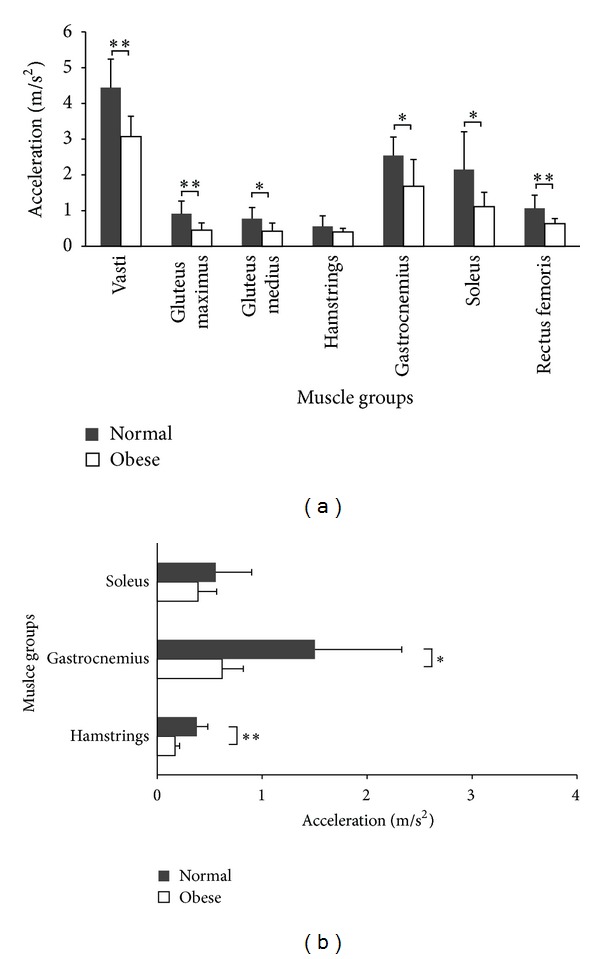
Comparison of peak vertical accelerations (a) and forward accelerations (b) of the major muscles between the OB group and the NW group.*P < 0.05, **P < 0.01.

Although the vertical acceleration of COM was nearly the same in both groups (Figure 1), individual muscle contributions differed (Figure 6). Nonobese children obviously had greater contributions to COM for almost all muscle groups due to a faster walking speed, except the contribution of hamstrings to vertical accelerations (*P* = 0.183) and the contribution of soleus to forward accelerations (*P* = 0.564). The magnitude of muscle activity generally increases with walking speed [15, 31]. Due to a slower gait speed, smaller muscle accelerations were observed in the OB group than in the NW group in this study. Liu et al. [15] also reported that larger vasti and gluteus maximus forces in the early stance and greater soleus and gastrocnemius forces in the late stance increased with walking speeds. The activity of gluteus medius in their results was considerably greater than ours. This suggested that lower hip abduction moment might be reproduced in our simulation results. The magnitude of the frontal hip joint moment was previously found slightly less than that of the hip flexor and knee extensor [23]. Thus, the movement of the hip abductor might be underestimated in our study, though data is not available at this time to confirm this suggestion.

### 3.4. Recommendations for Clinical Applications

Taking the joint reaction force and muscle function together, it is suggested that reduced walking speed is one of the strategies used by obese children to decrease the joint load and muscle requirement. Walking has been widely used in weight loss interventions for obese youth as a primary aerobic exercise modality [32]. According to our finding, self-selected speed walking should be more appropriate rather than fast walking as an exercise option for obese children. Although faster walking could consume more energy, it would increase the risk of musculoskeletal injury. Optimized mechanical efficiency and relatively lower joint loading during self-selected speed allow obese individuals  to exercise for longer time periods without fatigue and joint discomforts. Browning [33] also suggested that moderate speeds should be recommended as an appropriate form of exercise for obese adults and children without osteoarthritis or varus knee alignment to promote or maintain weight loss. However, if the misalignment or joint disorders have occurred, nonweight-bearing activities or weight-supported exercises should be used.

### 3.5. Limitation

We acknowledge that there are some limitations in our study. Firstly, musculoskeletal modeling requires accurate experimental marker placements. However, skin movement artifacts may affect the accuracy, especially on the hips. This may cause uncertainties in the determination of joint centers and then result in potential to over- or underestimated muscle activities. A second limitation is that the knee was modeled as a one-DOF planar joint. The varus or valgus movements cannot be investigated using this model. The actual frontal knee alignment of the subjects is impossible to match perfectly with this model. In fact, previous studies have shown that obese children had increased knee frontal plane moments during stance, which may distribute force across the medial or lateral compartment of the knee and create an increased risk of knee misalignment [24, 34]. This model error may reduce the activities of those muscles attached to the medial and lateral knee. Although our research did not aim to study the medial and lateral compartment of joint loads, this inaccuracy could result in potential over- or underestimated muscle activities, such as hip abductors. In addition, we did not measure EMG to verify the muscle excitations in obese children. This Hill-type muscle-based generic model has shown its reliability in various gait studies, but it is indeed possible that obese subjects stimulated slightly different muscle groups showing similar gait kinematics as normal weight subjects. Thus, conclusions for obese children based on simulations with current generic musculoskeletal parameters should be interpreted with caution.

### 3.6. Future Work

Given the prevalence of childhood obesity and the lack of specific musculoskeletal models for the obese population, it is critical that we continue to develop a  more accurate model to investigate the biomechanical effect of obesity at the musculoskeletal level. Use of medical imaging (e.g., MRI and DEXA) in the obese population will be extremely important to provide more accurate information on landmark positions, mass distribution of segments, and the muscle properties and then to further reduce the inaccuracy of dynamic simulation results. The combination of increased mass and potential altered joint axes is expected to be a contributor to long-term musculoskeletal injury and diseases in the obese populations and is a noteworthy area for future study. For the obese individual who already has an abnormal knee alignment (e.g., bowl legs, knock-kneed legs), a more complex knee model is needed to determine the gait mechanics with knee misalignments (varus/valgus). Researchers can further obtain the medial and lateral compartment joint load and the ligament forces by integrating them with medical imaging data. In addition, forward dynamic simulations have great potential in obesity prevention and intervention. In a recent review, Browning [33] pointed out that the utilization of complex individualized musculoskeletal models would allow us to predict the outcome of interventions. Based on an improved and verified “obese” musculoskeletal model, a computational framework can be developed to predict posttreatment outcome from pretreatment movement data for obese individuals. Dynamic simulations not only can assist specialists in designing targeted obesity treatments or exercise interventions but also can theoretically test the effectiveness of an orthopedic device (e.g., wedged insoles, knee varus/valgus brace) on musculoskeletal problems. The final challenge, similar to most muscle-driven simulations, is addressing muscle fatigue if using dynamic simulations to analyze exercise in obese children. Fatigue can limit the ability of a muscle to generate force and change the muscle activation characteristics [35, 36]. It will be necessary to develop a model that can be used in some situations where fatigue is likely to occur.

## 4. Conclusion

Obese children had a lower normalized compressive tibiofemoral force than nonobese children when walking at their self-selected speed. The vertical acceleration of COM was almost the same in the two groups, while the obese children had lower contributions of individual muscles to support and progression during gait. These findings suggested that obese children could adapt to a compensation  gait  strategy (e.g., reduced  walking  speed) to  avoid  increasing  joint  loads and muscle requirements during walking. The absolute compressive tibiofemoral force and muscle forces were still greater in obese children. The long-term biomechanical adaptations of the musculoskeletal system to accommodate the excess body weight are a concern.

## Figures and Tables

**Table 1:** Demographics of participants (mean ± standard deviation).

	Obese	Non-obese	*P*
Weight (kg)	75.1 ± 11.0	43.1 ± 10.5	**<0.001**
Height (cm)	155.4 ± 4.9	150.6 ± 6.0	0.103
Body mass index (kg/m^2^)	31.0 ± 3.4	18.8 ± 3.8	**<0.001**
Self-selected speed (m/s)	1.10 ± 0.08	1.22 ± 0.08	**0.014**

**Table 2:** Body weight normalized joint loading and muscle forces (mean ± standard deviation).

	Obese	Normal	*P*
CTF (×BW)	1.84 ± 0.24	2.49 ± 0.45	**0.003**
Hamstring (×BW)	0.47 ± 0.07	0.60 ± 0.07	**0.002**
Quadriceps (×BW)	0.98 ± 0.26	1.53 ± 0.52	**0.019**
Gastrocnemius (×BW)	0.38 ± 0.12	0.57 ± 0.08	**0.003**

CTF: compressive tibiofemoral force; BW: body weight.
